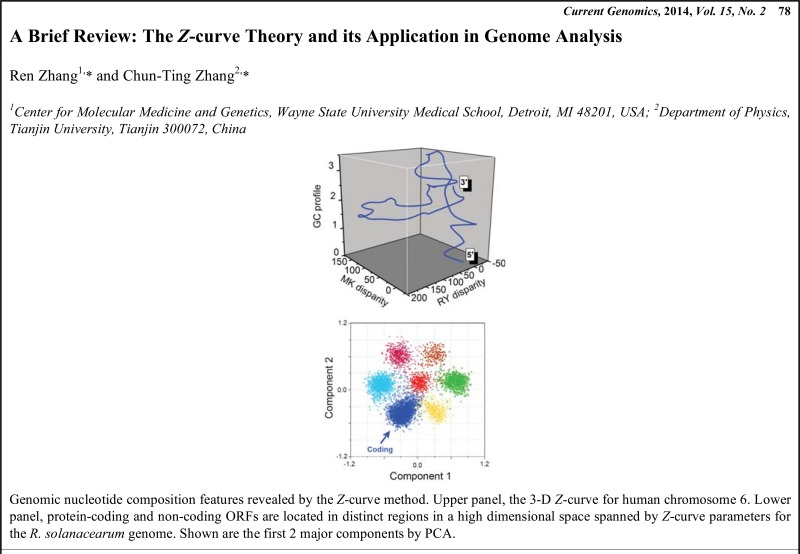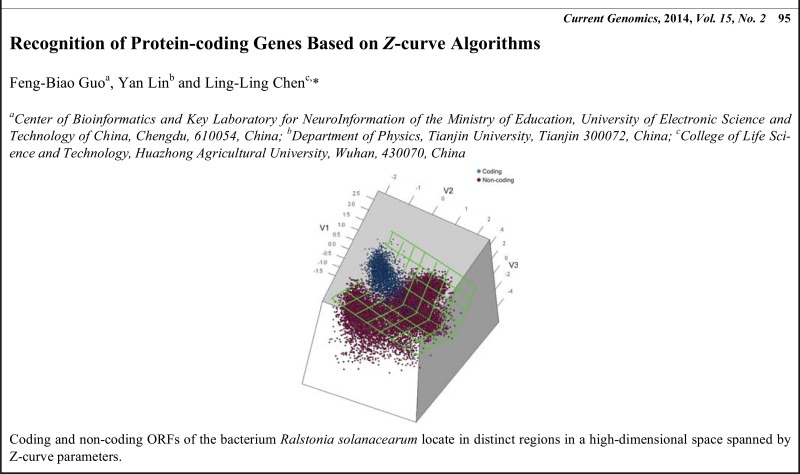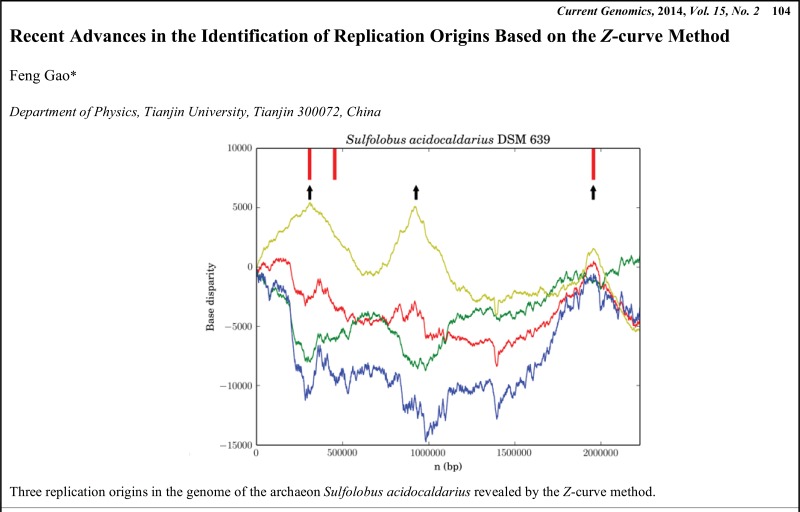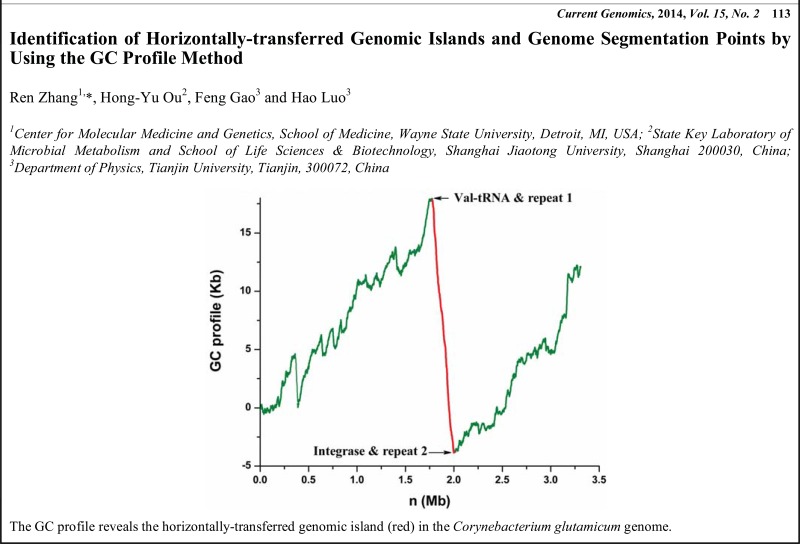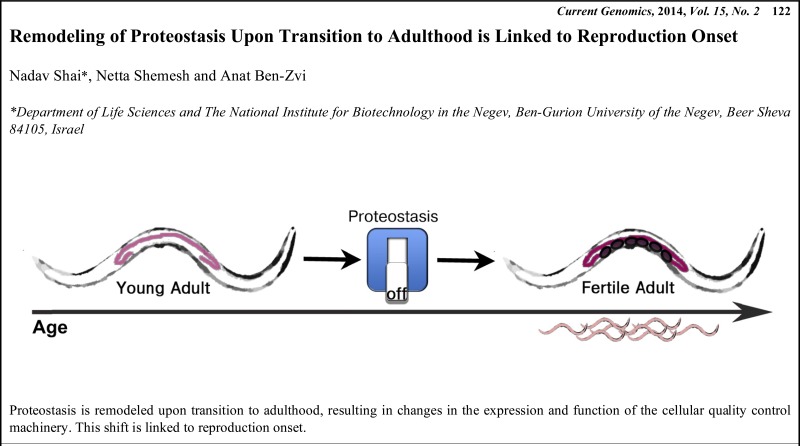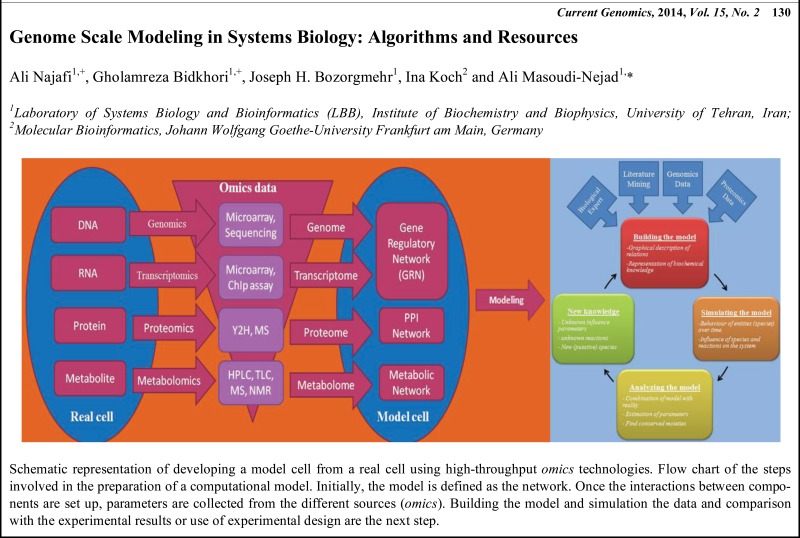# Graphical Abstracts

**DOI:** 10.2174/138920291502140421153813

**Published:** 2014-04